# Supplementary material for: Exploring Financial Challenges and University Support Systems for Student Financial Well-Being: A Scoping Review
Source: Int J Environ Res Public Health. 2025 Feb 27;22(3):356. doi: 10.3390/ijerph22030356 (PMC11941776; doi:10.3390/ijerph22030356)
Supplement: Supplementary file 1 [file ijerph-22-00356-s001.zip › ijerph-3487126-supplementary.pdf]

Supplementary Table S1: Reasons for exclusion of articles during screening

1. Articles on food insecurity training
2. Non-university student population
3. Protocol paper
4. Development of a poverty evaluation instrument for students
5. Staff interviews on their experiences dealing with food-insecure students
6. The use of single test scores as an outcome
7. Studies exploring campuses' procedures/policies without students' experience
8. Studies exploring entrepreneurship in students
9. Studies exploring food habits
10. Studies exploring poverty and other factors in relation to university education outside Australia
11. Studies exploring sex behaviors in students
12. Studies exploring antibiotic use in university students
13. Studies exploring climate change/food sustainability overseas
14. Studies exploring school curriculum
